# A Comparative Analysis of *Edwardsiella tarda*-Induced Transcriptome Profiles in RAW264.7 Cells Reveals New Insights into the Strategy of Bacterial Immune Evasion

**DOI:** 10.3390/ijms20225724

**Published:** 2019-11-15

**Authors:** Huili Li, Boguang Sun, Xianhui Ning, Shuai Jiang, Li Sun

**Affiliations:** 1 CAS Key Laboratory of Experimental Marine Biology, Center for Ocean Mega-Science, Chinese Academy of Sciences, Institute of Oceanology, 7 Nanhai Road, Qingdao 266071, China; lihuili17@mails.ucas.ac.cn (H.L.); sunboguang@qdio.ac.cn (B.S.); xhningouc@163.com (X.N.); sjiang@qdio.ac.cn (S.J.); 2 Laboratory for Marine Biology and Biotechnology, Qingdao National Laboratory for Marine Science and Technology, 1 Wenhai Road, Qingdao 266237, China; 3 College of Earth and Planetary Sciences, University of Chinese Academy of Sciences, 19 Yuquan Road, Beijing 100049, China

**Keywords:** *Edwardsiella tarda*, macrophage, infection, transcriptome, immune evasion

## Abstract

*Edwardsiella tarda* is a Gram-negative bacterial pathogen with a broad host range, including fish, reptiles, and mammals. One prominent virulence feature of *E. tarda* is its ability to survive and replicate in host phagocytes, but the relevant molecular mechanism is largely unknown. In this study, we examined the transcriptome profiles of RAW264.7 cells, a murine macrophage cell line, infected with live *E. tarda* or stimulated with dead *E. tarda* for 4 h and 8 h. Eighteen libraries were constructed, and an average of 69 million clean reads per library were obtained, with ~81.63% of the reads being successfully mapped to the reference genome. In total, 208 and 232 differentially expressed genes (DEGs) were identified between live and dead *E. tarda*-treated cells at 4 h and 8 h post-infection, respectively. The DEGs were markedly enriched in the Gene Ontology (GO) and Kyoto Encyclopedia of Genes and Genomes (KEGG) pathways associated with immunity. Live *E. tarda* differed strikingly from dead *E. tarda* in the regulation of immune related genes. Compared with dead *E. tarda*-treated cells, live *E. tarda*-treated cells exhibited marked and significant suppression in the induction of a large amount of immune genes, including RIG-I-like receptors, cytokines, and interferon-related genes. Furthermore, some of the immune genes highly regulated by live *E. tarda* formed complicated interaction networks with each other. Together, the results of this study revealed a transcriptome profile specifically induced by the active virulence elements of live *E. tarda* during the infection process, thus adding new insights into the intracellular infection mechanism of *E. tarda*. This study also provided a valuable set of target genes for further study of the immune evasion strategy of *E. tarda*.

## 1. Introduction

*Edwardsiella tarda* is a Gram-negative bacteria and a pathogen with a broad range of hosts, including fish, birds, reptiles, and mammals [1,2]. In aquaculture, *E. tarda* is a lethal pathogen that infects both freshwater and marine fish, and induces heavy economic losses by causing a severe systemic disease known as edwardsiellosis [2]. As a result, *E. tarda* is considered one of the most serious pathogens of aquaculture. In addition, *E. tarda* is the only species in the *Edwardsiella* genus that is pathogenic to humans [1]. In humans, *E. tarda* has been reported to cause gastroenteritis, septicemia, meningitis, colitis, liver cirrhosis, tuboovarian abscess, and sepsis [1,3,4].

Recently, many virulence-associated factors and systems have been identified in *E. tarda* [5,6,7]. Unlike most bacterial pathogens of aquaculture source, *E. tarda* exhibits a strong capacity to circumvent the antibacterial immune reactions of the host, which enables the bacteria to disseminate in host tissues and cause systemic infection. Accumulating evidence has indicated that *E. tarda* is able to survive and replicate in host serum and phagocytes, the latter including macrophages [8,9,10,11,12]. Macrophages are professional phagocytes that provide the first line of innate immune defense against invading pathogens [13]. Macrophages, as well as other types of phagocytes, remove pathogens via various means, notably lysosome-dependent bacterial destruction by acidic enzymes, production of reactive oxygen species (ROS) and reactive nitrogen, and secretion of antimicrobial factors that promote other immune cells to clear the pathogens [14,15,16]. To survive in phagocytes such as macrophages, pathogens have developed various strategies to avoid or eliminate the cellular killing effects [17,18]. For *E. tarda*, it is known to suppress ROS production in fish macrophages and inhibit apoptosis by manipulating the expression of apoptotic genes in a fish cell line [9,19]. In the murine macrophage cell line of RAW264.7 and bone marrow derived macrophages (BMDM), *E. tarda* employed the clathrin- and caveolin-mediated endocytosis pathways for cellular invasion and caused suicidal destruction of the host cells by triggering inflammasome and pyroptosis [20,21]. However, the molecular mechanism of *E. tarda* infection in phagocytes still remains to be investigated.

In this study, in order to gain new understanding of the cellular infection mechanism of *E. tarda*, we employed high-throughput sequencing technology to examine the global transcription profiles of RAW264.7 cells infected with live *E. tarda* or treated with dead *E. tarda* of different time points. Comparative transcriptome analyses were then conducted to identify differentially expressed genes between these groups. With this approach, we uncovered a large number of immune-related genes specifically induced by the active infection of live *E. tarda* rather than by the passive action of host cell phagocytosis against inactive *E. tarda*. The results of our study added new insights into the intracellular pathogenicity of *E. tarda* and provided valuable transcriptome data for future studies.

## 2. Results

### 2.1. Infection of E. tarda in RAW264.7 Cells

**Figure 1:**
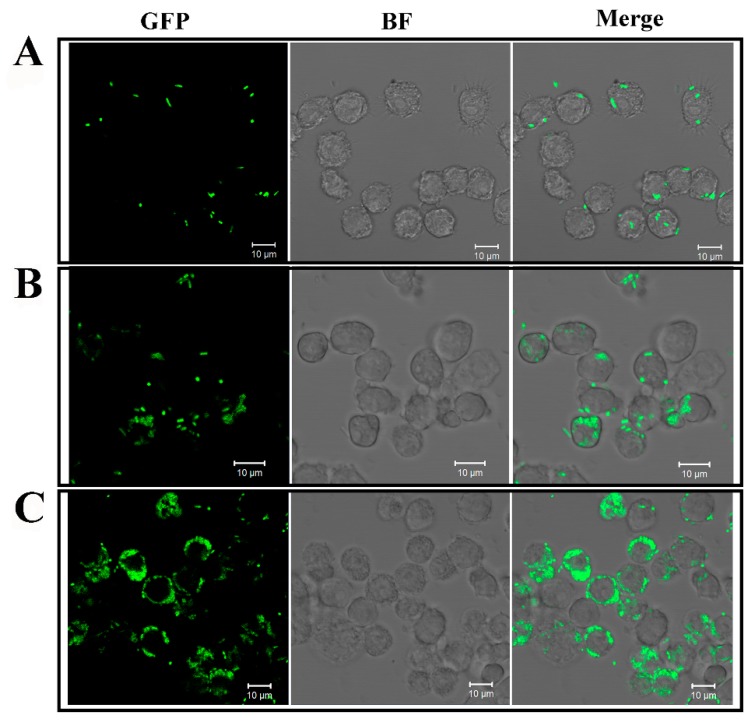
Microscopic observation of the intracellular replication of *Edwardsiella tarda* in RAW264.7 cells. RAW264.7 cells were infected with GFP-expressing *E. tarda* for 2 h. The extracellular and surface-attached bacteria were killed by antibiotic treatment. The cells were then incubated for 0 h (**A**), 4 h (**B**), and 8 h (**C**) to allow intracellular bacterial replication. After incubation at each time point, the cells were observed with a confocal microscope under bright field (BF) and fluorescent light (GFP). The merged image of each panel is shown on the right. Scale bar, 10 µm.

The results showed that in RAW264.7 cells infected with live *E. tarda*, the intracellular numbers markedly increased with time from 0 to 8 h (Figure 1). In contrast, in RAW264.7 cells similarly infected with dead *E. tarda*, phagocytosis of the bacteria into the cells was observed, however, no increase of intracellular bacterial number was detected (Figure S1). Untreated RAW264.7 cells showed no presence of bacteria (Figure S2).

### 2.2. RNA Sequencing and Read Mapping

The sequencing data of the 18 libraries are summarized in Table 1. A mean number of 69,507,649 filtered clean reads was obtained from each library, and 79.58–83.67% of the clean reads were mapped to the reference genome. The number of genes detected in each sample ranged from 12,605 to 12,891. Correlation analysis among sequencing samples showed good repeatability (Figure S3). All the sequencing data were submitted to Sequence Read Archive (SRA) in National Center for Biotechnology Information (NCBI) under the accession number PRJNA579883.

### 2.3. Differential Expression Analysis

**Figure 2:**
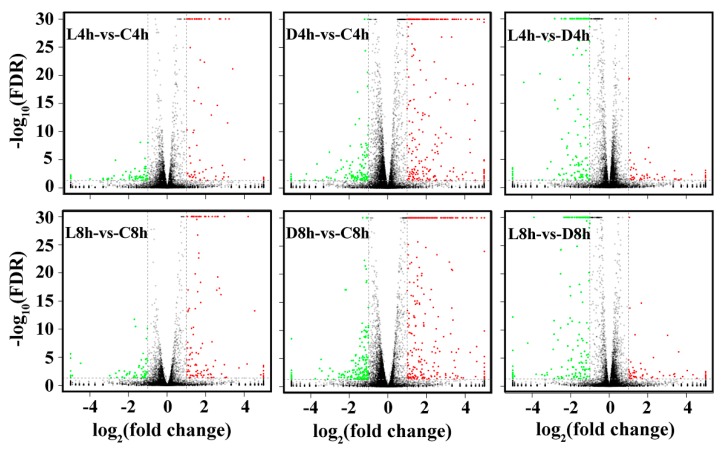
Volcano plot of differentially expressed genes (DEGs) between different groups of *Edwardsiella tarda*-treated RAW264.7 cells. Red, green, and black dots represent upregulated DEGs, downregulated DEGs, and non-DEGs, respectively. The X-axis indicates the logarithm of fold change. The Y-axis displays the negative logarithm to the base 30 of the t-test Q-values. C: control groups, D: dead *E. tarda*-treated groups, L: live *E. tarda*-treated groups; 4 h: 4 hpi; 8 h: 8 hpi.

As shown in Table 2 and Figure 2, the number of differentially expressed genes (DEGs) identified in L4h-vs-C4h was 145, with 81 being upregulated DEGs and 65 being downregulated DEGs. In contrast, the number of DEGs identified in D4h-vs-C4h was 387, with 268 upregulated DEGs and 119 downregulated DEGs. In the 8 hpi comparison, the number of DEGs identified in L8h-vs-C8h was 180, with 121 being upregulated DEGs and 59 being downregulated DEGs, while the number of DEGs in D8h-vs-C8h was 488, with 313 upregulated DEGs and 175 downregulated DEGs. When the live *E. tarda*-treated group and the dead *E. tarda*-treated group were compared, the number of DEGs in L4h-vs-D4h was 208, with 57 DEGs being upregulated and 151 DEGs being downregulated in the live *E. tarda*-treated group; the number of DEGs in L8h-vs-D8h was 232, with 63 DEGs being upregulated and 169 DEGs being downregulated in the live *E. tarda*-treated group (Table 2).

### 2.4. Validation of DEGs

**Figure 3:**
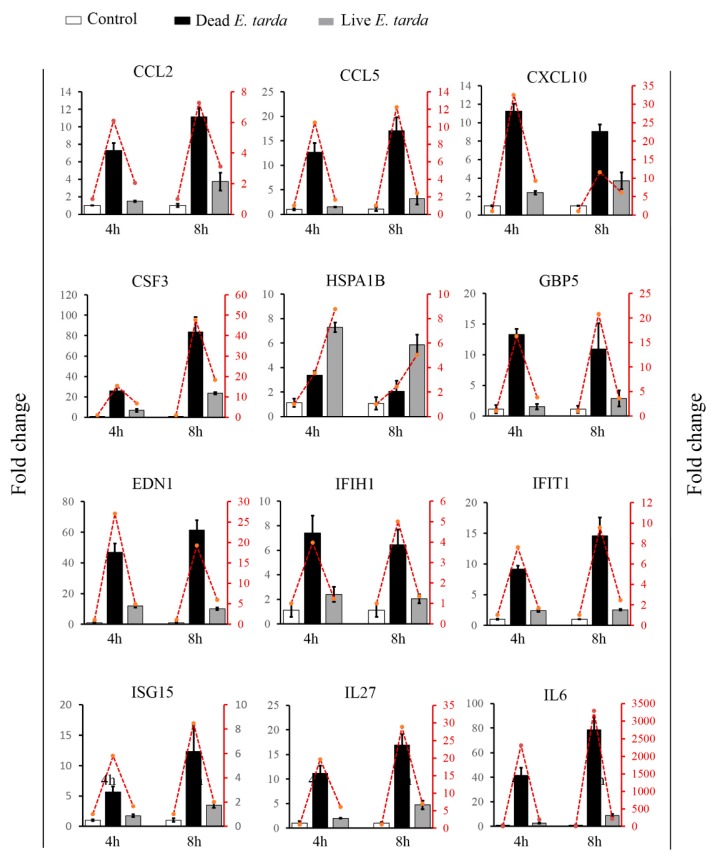
The expression patterns of 12 differentially expressed genes (DEGs) determined by qRT-PCR. RAW264.7 cells were treated with or without (control) dead or live *Edwardsiella tarda* for 4 h and 8 h, and the expressions of the 12 selected DEGs were determined by qRT-PCR. Values are the means of triplicate experiments and shown as means ± SEM. The histograms represent the results of qRT-PCR; the line charts represent the results of RNA-seq.

**Figure 4:**
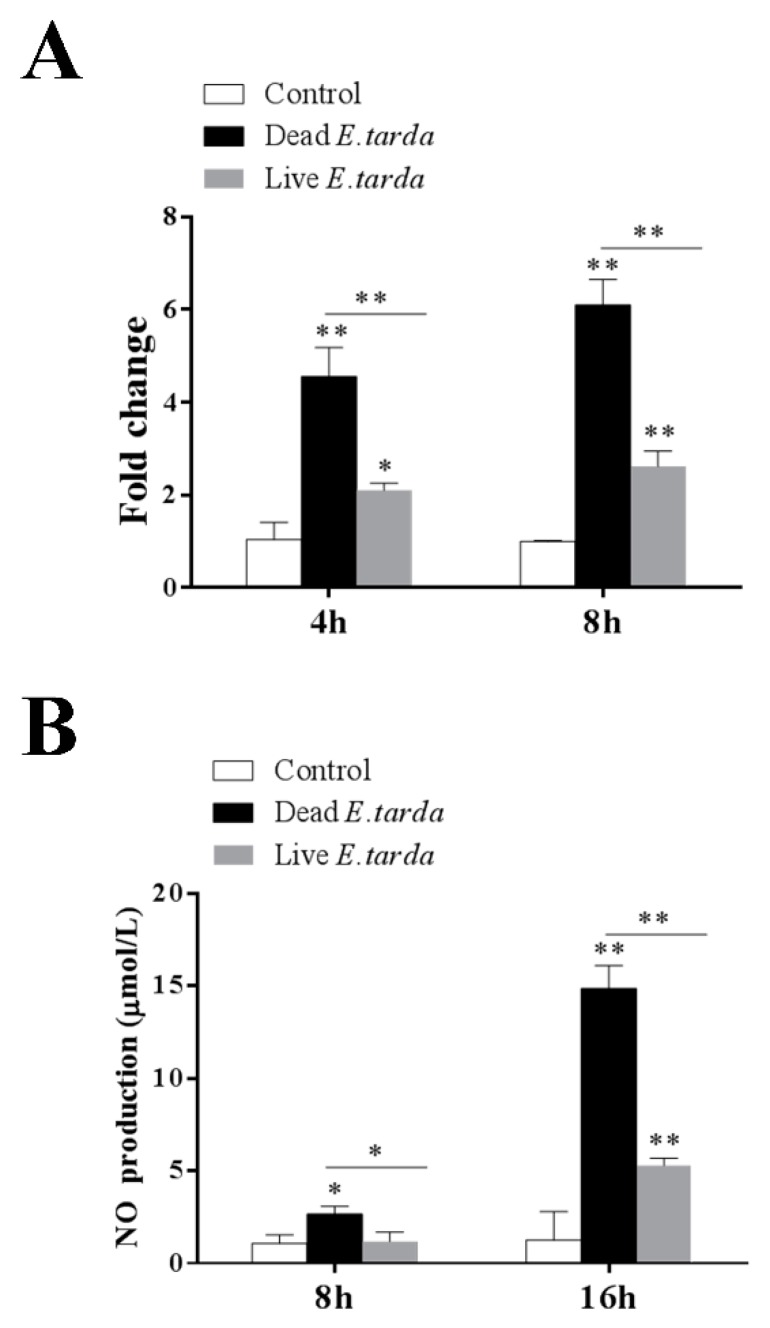
*NOS2* expression and nitric oxide (NO) production in RAW264.7 cells treated with *Edwardsiella tarda*. RAW264.7 cells were treated with or without (control) dead or live *E. tarda*. *NOS2* expression was determined at 4 h and 8 h by qRT-PCR (**A**), and NO level was determined at 8 h and 16 h by measuring nitrite (**B**). Values are the means of three replicates and shown as means ± SEM. * *p* < 0.05; ** *p* < 0.01.

In order to validate the RNA-seq results, the expression patterns of 12 DEGs were further analyzed with qRT-PCR. As shown in Figure 3, the qRT-PCR results of all examined DEGs were in good agreement with that of RNA-Seq. Furthermore, one of the DEGs identified by RNA-seq, i.e., *NOS2*, which catalyzes the production of nitric oxide (NO), was examined for both its expression based on qRT-PCR and its effect on NO production. The results showed that both dead and live *E. tarda* treatments significantly upregulated *NOS2* expression at 4 h and 8 h, however, the expression levels induced by live *E. tarda* were significantly lower than that induced by dead *E. tarda* (Figure 4A). These results were similar to that of RNA-seq. Consistently, in dead *E. tarda*-treated cells, NO production was significantly increased at both 8 h and 16 h, whereas in live *E. tarda*-treated cells, NO production was significantly increased only at 16 h and to a level that was significantly lower than that in dead *E. tarda*-treated cells (Figure 4B).

### 2.5. GO and KEGG Enrichment Analysis of the DEGs

**Figure 5:**
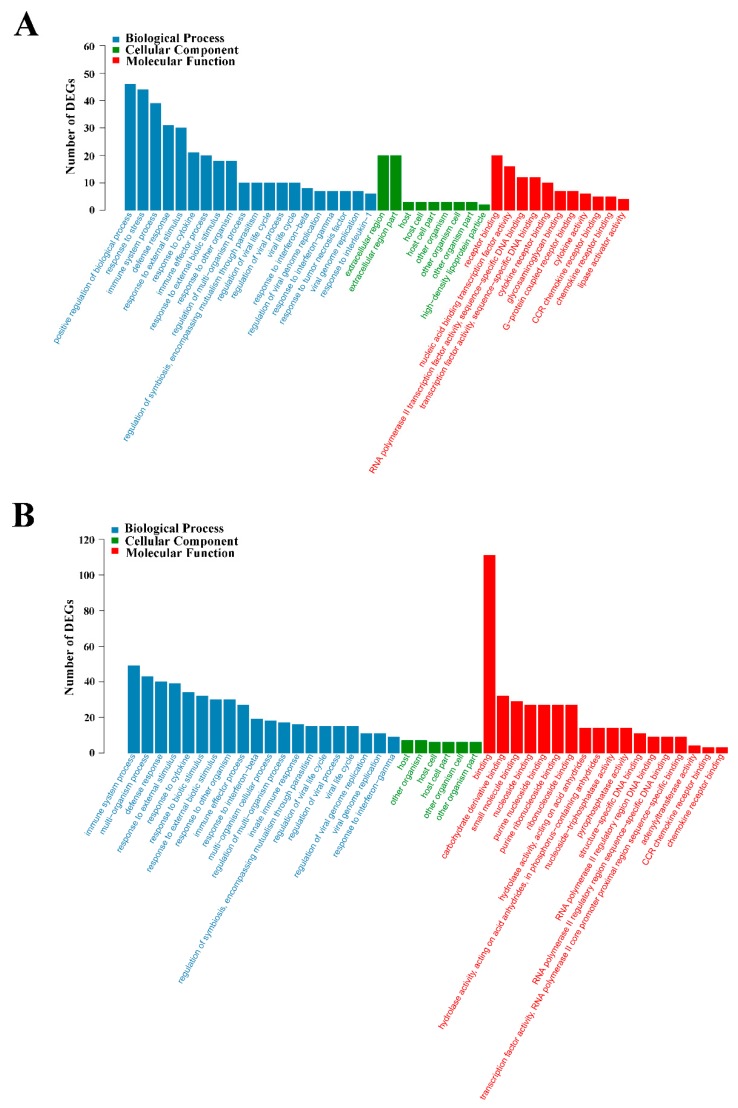
GO enrichment analysis of differentially expressed genes (DEGs) in L4h-vs-D4h (**A**) and L8h-vs-D8h (**B**). The X- and Y-axis represent the significantly enriched GO terms and the corresponding number of DEGs, respectively.

**Figure 6:**
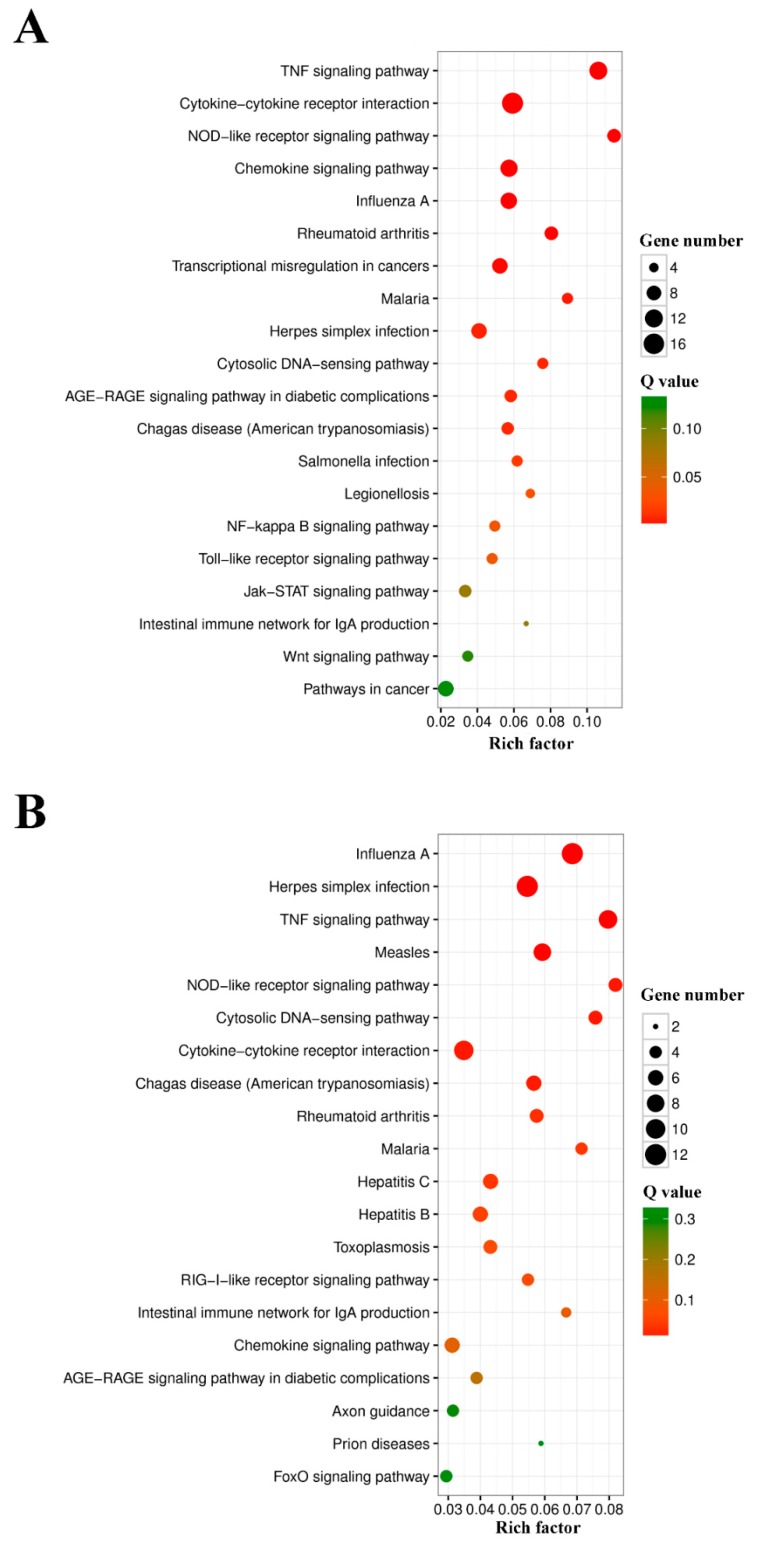
The top 20 enriched KEGG pathways in the differentially expressed genes (DEGs) of L4h-VS-D4h (**A**) and L8h-VS-D8h (**B**). The color and size of the dots indicate Q-values and DEG numbers in pathways, respectively.

GO functional enrichment analysis indicated that the DEGs of L4h-vs-D4h and L8h-vs-D8h were classified into three categories: Biological Process, Cellular Component, and Molecular Function. The complete or the top 20 significantly enriched GO terms of the three categories are shown in Figure 5. In the DEGs of L4h-vs-D4h, the GO terms of positive regulation of biological process, response to stress, immune system process, defense response, response to external stimulus, response to cytokine, immune effector process, response to external biotic stimulus, and response to another organism, were highly represented in the category of Biological Process. The GO terms of extracellular region and extracellular region part were highly represented in the category of Cellular Component. In the category of Molecular Function, the GO terms of receptor binding, nucleic acid binding transcription factor activity, cytokine receptor binding, cytokine activity, CCR chemokine receptor binding, and chemokine receptor binding, were also significantly enriched (Figure 5A). In the DEGs of L8h-vs-D8h, the GO terms of immune system process, multi−organism process, defense response, response to external stimulus, response to cytokine, response to biotic stimulus, response to external biotic stimulus, response to another organism, and immune effector process were highly represented in the Biological Process category. The GO terms of host, other organism, host cell, host cell part, other organism cell, and other organism part were significantly enriched in the Cellular Component category. In the category of Molecular Function, the GO term of binding was dominant. CCR chemokine receptor binding and chemokine receptor binding were also significantly enriched in the category of Molecular Function (Figure 5B). In KEGG analysis, the top 20 enriched pathways are shown in Figure 6. In the DEGs of L4h-vs-D4h, the top most represented KEGG categories included TNF signaling pathway, cytokine-cytokine receptor interaction, NOD-like receptor signaling pathway, chemokine signaling pathway, influenza A, rheumatoid arthritis, transcriptional misregulation in cancers, malaria, and herpes simplex infection. Other significantly enriched pathways included cytosolic DNA-sensing pathway, AGE-RAGE signaling pathway in diabetic complications, chagas disease (American trypanosomiasis), salmonella infection, legionellosis, NF-kappa B signaling pathway, and toll-like receptor signaling pathway (Figure 6A). In the DEGs of L8h-vs-D8h, the top most represented KEGG categories included influenza A, herpes simplex infection, TNF signaling pathway, measles, NOD-like receptor signaling pathway, cytosolic DNA sensing pathway, cytokine-cytokine receptor interaction, chagas disease (American trypanosomiasis), and rheumatoid arthritis. Other significantly enriched pathways included malaria, hepatitis C, and hepatitis B (Figure 6B).

### 2.6. DEGs Involved in the Immune Response Induced by Live E. tarda

Diverse immune-related DEGs were identified in L4h-vs-D4h and L8h-vs-D8h, including retinoic acid-inducible gene I (RIG-I)-like receptors (RLRs), cytokines, interferon-related genes, and other immune-related genes, most of which were strikingly downregulated in live *E. tarda*-treated groups compared with that in dead *E. tarda*-treated groups (Table 3). For the RIG-I-like receptors, significant regulation was found in the genes of interferon induced with helicase C domain 1 (*IFIH1*), DEAD box polypeptide 58 (*DDX58*), and DEXH box polypeptide 58 (*DHX58*). *IFIH1* was downregulated at both L4h-vs-D4h and L8h-vs-D8h, while *DDX58* and *DHX58* were downregulated at L8h-vs-D8h. A large number of DEGs in the category of cytokines were identified, including interleukins, chemokines, and colony stimulating factor. Interleukin (IL)-6 (*IL-6*), *IL-27*, *IL1F9*, chemokine C-C motif ligand (*CCL*) *5*, *CCL7*, *CCL2*, and colony stimulating factor 3 (*CSF3*) were downregulated in both L4h-vs-D4h and L8h-vs-D8h. Fifteen interferon-related DEGs were detected, among which, guanylate binding protein (*GBP*) *2*, *GBP5*, *GBP2b*, interferon-induced protein with tetratricopeptide repeats (*IFIT*) *3*, *IFIT3b*, *IFIT1*, ubiquitin-like modifier (*ISG15*), immunity-related GTPase family M member 1(*IRGM1*), and *IRGM2* were downregulated in both L4h-vs-D4h and L8h-vs-D8h. Other immune-related DEGs included myristoylated alanine rich protein kinase C substrate (*MARCKS*), programmed cell death 1 (*PDCD1*), inducible nitric oxide synthase 2 (*NOS2*), heat shock protein 1B (*HSPA1B*), endothelin 1 (*EDN1*), signal transducer and activator of transcription 2 (*STAT2*), and complement component 5a receptor 1 (*C5AR1*). Among these genes, *MARCKS* and *EDN1* were downregulated in both L4h-vs-D4h and L8h-vs-D8h.

### 2.7. The Interaction Networks of Immune-Related DEGs

**Figure 7:**
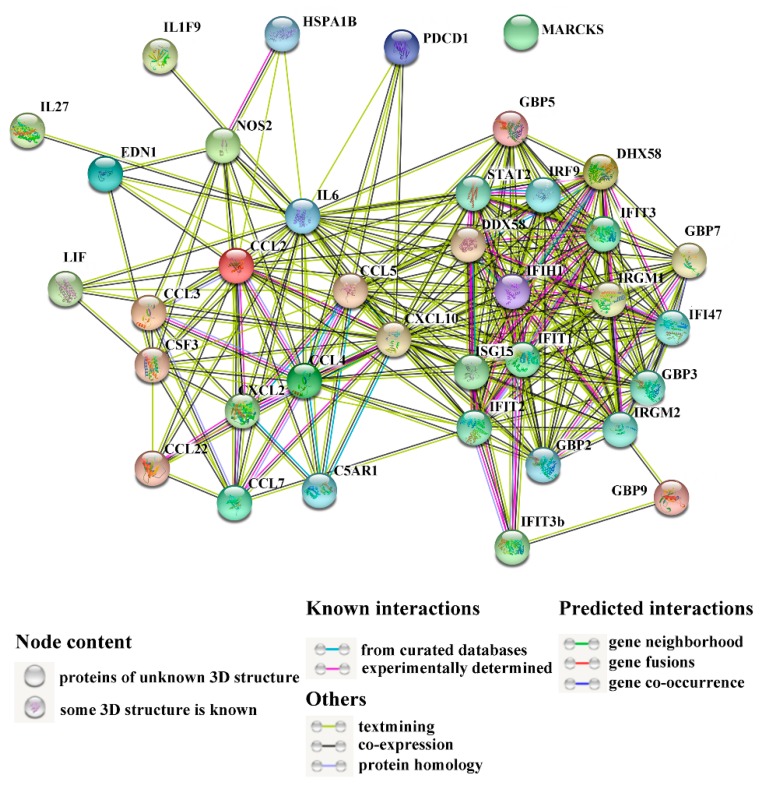
The interaction networks of differentially expressed genes (DEGs) associated with immunity. Network nodes represent the proteins of immune-related DEGs; lines indicate association between the linked DEGs.

The interaction networks of immune-related DEGs are shown in Figure 7. Table 4 lists the top 10 key DEGs with multiple interaction relationships. Among these DEGs, chemokine C-X-C motif ligand (*CXCL*) *10* displayed the highest number (29) of interactions. Next to *CXCL10* was *IL6*, which interacted with 28 DEGs. Other highly interactive DEGs included *IFIT2*, *CCL5*, *ISG15*, *IFIH1*, *IFIT1*, *CCL2*, *DDX58*, and *IRGM1*, which exhibited interaction numbers ranging from 18 to 23 (Table 4).

### 2.8. Phosphorylation Status of NF-κB in RAW264.7 Cells Infected with Live and Dead E. tarda

**Figure 8:**
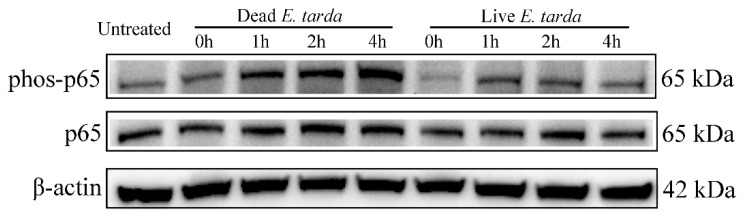
Effects of live and dead *Edwardsiella tarda* on NF-κB p65 phosphorylation. RAW264.7 cells were infected with or without live or dead *E. tarda* for 2 h and then treated with 100 µg/mL gentamicin for 1 h. The cells were washed and incubated in fresh Opti-MEM containing 20 µg/mL gentamicin for 0 h, 1 h, 2 h or 4 h. After incubation, phosphorylation of NF-κB p65 (phos-p65) was detected by Western blot. β-actin was used as an internal reference.

To examine whether live and dead *E. tarda* infection induced different response with respect to NF-κB activation, the phosphorylation status of NF-κB p65 in RAW264.7 cells treated with live and dead *E. tarda* for different time was examined. The results showed that in RAW264.7 cells infected with dead *E. tarda*, phosphorylation of NF-κB p65 increased with time, while in RAW264.7 cells infected with live *E. tarda*, phosphorylation of NF-κB p65 was comparable during the course of infection and at each time point was apparently lower in level than that in dead *E. tarda*-infected cells (Figure 8).

## 3. Discussion

Survival in host phagocytic cells is the most important virulence characteristic of intracellular bacteria [22]. In this study, we observed that following incubation of *E. tarda* with RAW264.7 cells, the number of intracellular bacteria increased with time, indicating an ability of *E. tarda* to evade the bactericidal activities of macrophages and replicate inside phagocytic cells. To examine the host cell response triggered specifically during the process of *E. tarda* infection, we compared the transcriptomes of RAW264.7 cells exposed to live and dead *E. tarda*. Our results revealed that live and dead *E. tarda* elicited markedly different cellular responses. A previous study with human primary macrophages showed that the cells treated with inactivated *Mycobacterium tuberculosis* exhibited more differentially expressed microRNAs than the cells treated with live *M. tuberculosis* [13]. In our study, we found that compared with live *E. tarda*, dead *E. tarda* caused a much stronger host response, both in the number of the DEGs and in the degree of the expressional change of the DEGs. The DEGs induced by dead *E. tarda* were likely involved in innate immunity, particularly in that related to phagocytosis, while the DEGs specifically induced by live *E. tarda* were at least in part involved in the active immune evasion of the pathogen. GO annotation and KEGG pathway analysis classified immune-related DEGs into several categories, of which the DEGs strongly regulated by live *E. tarda* are discussed below.

### 3.1. RIG-I-Like Receptors

RIG-I-like receptors (RLRs) are a type of pattern-recognition receptors (PRRs) that identify pathogen association molecular patterns (PAMPs) and activate non-specific host defenses [23]. RLRs are cytosolic RNA-sensing proteins responsible for intracellular immune surveillance against primarily viral infections [24]. Currently, the RLRs family contains three members, i.e., *IFIH1*, *DDX58* and *DHX58*. Interestingly, in our study, all the three RLRs members were down-regulated significantly in live *E. tarda*-infected groups compared with that in dead *E. tarda*-treated groups. In addition, *IFIH1* and *DDX58* were among the top 10 key genes based on protein-protein interaction analysis. Previous studies showed that *DDX58* and *IFIH1* participated in the production of type I interferon (IFN) and the intracellular immunity against various bacteria including *Escherichia coli*, *Acinetobacter baumannii*, *Legionella pneumophila*, and *Pseudomonas aeruginosa* [25,26,27]. The role of *DHX58* in antimicrobial immunity is less clear, but it has been demonstrated that *DHX58* could facilitate viral RNA recognition by *IFIH1* and *DDX58* [28]. Based on these observations, the systematic down-regulation of all RLR members by live *E. tarda* observed in our study suggested an important role of RLRs in *E. tarda* infection. It is likely that RLRs, as intracellular PRRs, recognize *E. tarda*-associated PAMPs during the intracellular infection process of the pathogen and elicit cellular responses that promote *E. tarda* clearance. As such, the downregulation of the expressions of the PRRs represents an infection strategy of *E. tarda* to overcome the immune defense of the host cells.

### 3.2. Cytokines

Cytokines are secreted mainly by activated immune-related cells and vital to antimicrobial infections [29,30]. In our study, it was found that compared with dead *E. tarda*, live *E. tarda* significantly down-regulated the expression of a number of interleukins and chemokines. Among the interleukins, *IL6* was the most down-regulated, with a more than 10-fold change in expression level at both 4 hpi and 8 hpi. In addition, *IL6* was a top 10 key DEGs with extensive protein-protein interactions. *IL6* is known to be one of the major pro-inflammatory cytokines and participate in the infection process of bacterial pathogens [31,32]. A previous study showed that *Mycobacterium marinum* suppressed the production of IL6 in human macrophages to facilitate its survival [33]. In our study, it is very likely that by down-regulating *IL6* expression, *E. tarda* blocked the induction of inflammation response in RAW264.7 cells, which allowed the pathogen to survive inside the macrophages.

Chemokines are classified into four families, i.e., XC, CC, CXC, and CX3C, of which, CXC and CC chemokines are the two major families [34]. During immune surveillance, chemokines guide cells of the immune system to the sites of infection and are important for effective clearance of pathogens [35,36,37,38]. In our study, comparing with dead *E. tarda*, live *E. tarda* significantly down-regulated the expressions of eight chemokines, all of which are of the CC and CXC families, suggesting an extensive regulatory effect of *E. tarda* on the major groups of chemokines. Among these differentially expressed chemokine genes, *CXCL10*, *CCL5*, and *CCL2* were identified to be the key genes with multiple interaction relationships with other genes. These results underlay the importance of CC and CXC chemokines in host immune defense, which makes them one of the major targets of manipulation by *E. tarda*.

### 3.3. Interferon-Related Genes

Interferons (IFNs) are potent inducers of antimicrobial effectors and essential to host defense against intracellular pathogens [39,40,41,42]. In our study, a series of interferon-related genes were found to be down-regulated in live *E. tarda*-treated groups compared with that in dead *E. tarda*-treated groups. Strikingly, six members of the GBP family were significantly down-regulated by live *E. tarda*, with *GBP5* being the most down-regulated. GBPs are known to participate in inflammasome assembly, apoptosis, and pyroptosis, and play a vital role in the defense against vacuolar pathogens [43,44,45]. The systematic downregulation of a large number of GBPs stressed the importance of GBP-mediated immunity in the clearance of *E. tarda*. Other strikingly suppressed genes included the family of IFIT, in which four members were significantly down-regulated by live *E. tarda*. IFITs are known to participate in antiviral immunity, but their functions in bacterial infection are essentially unknown [46]. In our study, the IFITs were not only down-regulated but also among the top 10 key genes with multiple interactive relationships with other genes. These observations indicated a potentially profound effect of IFITs on *E. tarda* infection. Other interferon-related DEGs down-regulated at both 4 hpi and 8 hpi included two members of the immunity-related GTPase (IRG) family, which is known to facilitate resistance against intracellular bacteria [47,48], and *ISG15*, which encodes an ubiquitin-like protein and is involved in viral and bacterial infections [49,50,51]. The significant downregulation of these genes by live *E. tarda* suggested that they likely played a vital role in the intracellular infection of *E. tarda*.

### 3.4. Other Immune-Related Genes

*NOS2* regulates the production of NO, an important component of host defense against intracellular pathogens [52,53]. In order to survive, some pathogens have evolved ways of avoiding NO-mediated killing [54,55,56,57]. Previous studies showed that *E. tarda* induced NO production in RAW264.7 cells [11]. Consistently, in our study, the levels of *NOS2* mRNA and NO production were augmented by both live and dead *E. tarda*, however, the folds of augmentation induced by live *E. tarda* were significantly lower than that induced by dead *E. tarda*. These results indicated that live *E. tarda* was able to inhibit, though not completely, NO-mediated immune response at the transcription level. Other immune-related DEGs included *EDN1*, a pro-inflammatory mediator known to play a role in *Mycobacterium tuberculosis* infection [58,59], and *MARCKS*, a lipopolysaccharide-induced protein kinase C substrate. *MARCKS* has been proposed to regulate actin-membrane interactions and is involved in phagocytosis and membrane trafficking [60,61]. The downregulation of these genes by live *E. tarda* may reduce inflammation and phagocytosis associated anti-bacterial effect, thus facilitating the invasion and survival of the pathogen.

NF-κB is a vital immune regulator, which can be activated by RIG-I signaling and activates the transcription of a large array of cytokines and other immune genes, such as *NOS2* [62,63,64]. In the present work, we observed that the phosphorylation level of NF-κB p65 was decreased upon live versus dead *E. tarda* infection, indicating a suppression of NF-κB activity by live *E. tarda*. In consistence, members of RLRs (*IFIH1*, *DDX58* and *DHX58*) and cytokines (such as *IL6*, *CCL2*, and *CCL5*), as well as *NOS2*, which are known to be the upstream PRRs and downstream target genes of NF-κB, respectively, were downregulated in RAW264.7 cells infected by live *E. tarda*. These observations implythat *E. tarda* is likely able to modulate the transcription of the PRRs of the RIG-I pathway and retard the subsequent activation of NF-κB signaling, thereby subverting host immune responses and facilitating infection.

In conclusion, in this study, we identified a large number of immune genes, in particular RIG-I-like receptors, cytokines, and interferon-regulated genes, associated with the intracellular infection of *E. tarda*. Since these genes were identified based on a comparative analysis between dead and live *E. tarda*-induced transcriptomes, they primarily represent the genes specifically induced by the virulence determinants of *E. tarda* expressed during the active infection process. Hence, these DEGs are very likely the manipulation targets of *E. tarda* immune evasion. Our results added new insights into the intracellular infection mechanism of *E. tarda* and provided valuable targets for future studies of *E. tarda*-host interaction.

## 4. Materials and Methods

### 4.1. Bacterial Strains and Cell Culture

The *E. tarda* strain used in this study was a fish pathogen that has been reported previously [65]. *E. tarda* containing the GFP-expressing plasmid pGFPuv has been reported previously [20]. The GFP- expressing *E. tarda* was used for confocal microscopy described below. *E. tarda* was cultured in Opti-MEM (Gibco, Grand Island, NY, USA) without shaking at 30 °C. RAW264.7 cells were purchased from American Tissue Culture Collection (ATCC, Rockville, MD, USA). The cells were cultured in DMEM supplemented with 10% fetal bovine serum (FBS) (Gibco, Grand Island, NY, USA) at 37 °C in a humidified atmosphere containing 5% carbon dioxide.

### 4.2. Cellular Infection and Confocal Microscopy

RAW264.7 cells were infected with *E. tarda* as described previously [20] with slight adjustments. Briefly, *E. tarda* was grown in Opti-MEM (Gibco, USA) at 30 °C without shaking to an OD_600_ of 0.6. To prepare live *E. tarda* sample, the bacteria were collected by centrifugation at 8000× *g*, washed with PBS, and resuspended in Opti-MEM to 1 × 10^8^ CFU/mL. To prepare inactivated *E. tarda* samples, the bacteria were heated at 65 °C for 45 min, and loss of bacterial viability was confirmed by plating onto Luria-Bertani (LB) agar plates and failing to see any bacterial growth. After heat inactivation, the bacteria were collected by centrifugation, washed with PBS, and resuspended in Opti-MEM as above. Cellular infection was conducted in triplicate as follows. Equal amounts of live and inactivated *E. tarda* were added separately to RAW264.7 cells in a 24-well plate at a multiplicity of infection (MOI) of 10:1. The control group of RAW264.7 cells was added with an equal amount of Opti-MEM. The plate was centrifuged at 400× *g* for 10 min, followed by incubation at 30 °C for 2 h (h). After incubation, the supernatant of the culture was removed. To kill extracellular *E. tarda*, fresh Opti-MEM containing gentamicin (100 µg/mL) was added to the plate, and the plate was incubated at 30 °C for 1 h. The cells were then washed three times with PBS and cultured in Opti-MEM containing 20 µg/mL gentamicin for 4 h or 8 h to allow intracellular replication of the bacteria. The cells were then used for RNA sequencing (described below) and observation with a Zeiss LSM 710 confocal microscope (Carl Zeiss, Oberkochen, Germany). For microscopic observation of dead *E. tarda*-treated RAW264.7 cells, *E. tarda* was inactivated as above, washed with PBS, and treated with 1 mg/mL fluorescein isothiocyanate (FITC) (Tiangen, Beijing, China) at 37 °C with shaking for 1.5 h. The FITC-labeled dead *E. tarda* was extensively washed with PBS and then used for cellular infection as above.

### 4.3. RNA Preparation, Library Construction, and Sequencing

RAW264.7 cells were treated with live *E. tarda* for 4 h and 8 h (named L4h and L8h, respectively) or with inactivated (dead) *E. tarda* for 4 h and 8 h (named D4h and D8h, respectively). As controls, RAW264.7 cells without any bacterial treatment were similarly cultured for 4 h and 8 h (named C4h and C8h, respectively). Triplicate samples of the above RAW264.7 cells were collected and used for the construction of 18 libraries. Total RNA extraction was conducted using Trizol reagent (Invitrogen, Carlsbad, CA, USA). The quality of the purified RNA was assessed using Agilent 2100 Bioanalyzer. RNA samples with high integrity (RIN > 8.0) and concentration of above 200 ng/µl were used for cDNA library construction. mRNA was enriched with Oligo (dT) beads (Qiagen, Hilden, Germany). The enriched mRNA was fragmented into short fragments and reverse transcribed into first-strand cDNA. The second-strand cDNA was synthesized using DNA polymerase I (Thermo Scientific, Waltham, MA, USA) and dNTP in the presence of RNase H. The cDNA fragments were purified with QiaQuick PCR extraction kit (Qiagen, Hilden, Germany), end repaired, poly (A) added, and ligated to Illumina sequencing adapters. The cDNA with the size of approximately 200 bp was selected and paired-end (PE150) sequenced by Illumina HiSeq^TM^ 2500 platform in Gene Denovo Biotechnology Co (Guangzhou, China).

### 4.4. Sequence Quality Control and Data Processing

Raw reads were filtered by removing the low quality reads, including reads containing adapters, reads containing nucleotide with q quality score lower than 20, and reads with unknown nucleotides larger than 10%, before mapping to ribosome RNA (rRNA) database in Bowtie 2 version 2.2.8 [66]. The rRNA-mapped reads were removed, and the remaining reads were mapped to the *Mus musculus* reference genome (https://www.ncbi.nlm.nih.gov/genome/52?genome_assembly_id=334509) using TopHat v2.1.1 [67]. The reconstruction of the transcripts was conducted using software cufflinks v2.2.1 [68]. Gene abundances were quantified with software RSEM v1.2.19 [69]. The gene expression level was normalized by using the Fragments Per Kilobase of transcript per Million mapped reads (FPKM) method. Correlation analysis among sequencing samples were performed with R package gmodels (http://www.r-project.org/).

### 4.5. Differentially Expressed Genes (DEGs) Analysis

To examine the differentially expressed genes (DEGs) between different groups, pairwise comparisons of the DEGs among the different groups were carried out, i.e., live *E. tarda*-treated groups versus control groups at 4 hpi and 8 hpi (L4h-vs-C4h and L8h-vs-C8h, respectively), dead *E. tarda*-treated groups versus control groups at 4 hpi and 8 hpi (D4h-vs-C4h and D8h-vs-C8h, respectively), and live *E. tarda*-treated groups versus dead *E. tarda*-treated groups at 4 hpi and 8hpi (L4h-vs-D8h and L8h-vs-D8h, respectively). DEGs were identified using the edgeR package (http://www.r-project.org/). Genes with a fold change ≥ 2 and a false discovery rate (FDR) < 0.05 were treated as significant DEGs.

### 4.6. Gene Ontology (GO) and Kyoto Encyclopedia of Genes and Genomes (KEGG) Enrichment Analysis

DEGs were further annotated by GO functional enrichment and KEGG pathway analysis. All DEGs were mapped to the GO terms in the Gene Ontology database (http://www.geneontology.org/), and gene numbers were calculated for every term. Pathway enrichment analysis was performed using the Kyoto Encyclopedia of Genes and Genomes database [70]. After multiple test correction, GO terms and pathways with Q value < 0.05 were considered to be significantly enriched in DEGs.

### 4.7. Validation of DEGs by Quantitative Real-Time Reverse Transcription-PCR (qRT-PCR)

The sequences of the primers used for qRT-PCR are listed in Table 5. The mRNA prepared in the above cellular infection was used for cDNA synthesis with RevertAid First Strand cDNA Synthesis Kit (Thermo Scientific, Waltham, MA, USA). qRT-PCR was carried out with Eppendorf Mastercycler epgradient S (Eppendorf, Hamburg, Germany) using TB Green^®^ Premix Ex Taq™ II (Takara, Dalian, China). The PCR reaction was performed in a 20 μL volume containing 10 μL TB Green Premix Ex Taq II, 0.4 μM specific forward primer and reverse primer, and 2.0 μL diluted cDNA (50 ng/μL). The PCR conditions were 95 °C for 30 s, followed by 40 cycles of 95 °C for 5 s and 60 °C for 30 s. The specificity of qRT-PCR products was examined by melting curve analysis. The expression of each gene was normalized to that of glyceraldehyde-3-phosphate dehydrogenase (GAPDH) and calculated using the comparative threshold cycle method (2^−ΔΔCT^). The experiment was performed in triplicate.

### 4.8. Determination of NOS2 Expression and Nitric Oxide (NO) Production

RAW264.7 cells were treated with or without (control) dead or live *E. tarda* as described above for 4 h, 8 h, and 16 h. NOS2 expression was determined by qRT-PCR as described above. NO production was determined by measuring its end product nitrite using the Nitric Oxide Assay Kit (Beyotime, Beijing, China) according to the manufacturer’s instructions. The assays were performed three times.

### 4.9. Construction of Protein-Protein Interaction Networks

The immune-related DEGs in Table 3 were used to construct protein-protein interaction (PPI) networks. PPI networks were constructed using STRING v10.0 (http://string-db.org/) with default parameters [71].

### 4.10. Western Blot to Detect Phospho-NF-κB p65

RAW264.7 cells were treated with dead or live *E. tarda* as above for 0 h, 1 h, 2 h, and 4 h. The cells were lysed on ice for 30 min with RIPA lysis buffer (Beyotime, Beijing, China) containing phosphatase inhibitor cocktail (Beyotime, Beijing, China). The cell lysates were mixed with SDS-PAGE loading buffer and boiled at 100 °C for 10 min. The samples were then subjected to SDS-PAGE, and the separated proteins were electro-transferred from gels to nitrocellulose blotting membranes (GE healthcare, Germany). The membranes were soaked in TBST (20 mM Tris, pH 7.5; 500 mM NaCl; 0.1% Tween 20) containing 5% bovine serum albumin (Solarbio, Beijing, China) for 2 h. The membranes were incubated with rabbit anti-phospho-NF-κB p65 (Ser536) monoclonal antibody (Cell Signaling Technology, Beverly, MA, USA), anti-NF-κB p65 monoclonal antibody (ABclonal, Wuhan, China), or anti-β-actin monoclonal antibody (ABclonal, Wuhan, China) at 4 °C for overnight. After extensive washing with TBST, the membranes were incubated with HRP-conjugated anti-rabbit antibody (Abcam, Cambridge, UK) for 1h at room temperature. The membranes were washed with TBST for three times and incubated with enhanced chemiluminescence (ECL) solution (Beyotime, Beijing, China). The membranes were visualized using a GelDoc XR System (Bio-Rad, Hercules, CA, USA).

### 4.11. Statistical Analysis

All experiments were performed three times. Statistical analyses were performed using the SPSS 17.0 software (SPSS Inc., Chicago, IL, USA). Data were analyzed with Student’s *t*-test, and statistical significance was defined as *p* < 0.05.

## Figures and Tables

**Table 1:** Summary of the sequencing data. C, control groups; D, dead *E. tarda*-treated groups; L, live *E. tarda*-treated groups; 4 h, 4 h post infection (hpi); 8 h, 8 hpi. Each sample was triplicated as indicated by the number after the hyphen, e.g., C4h-1, C4h-2, and C4h-3.

Samples	Read Length (bp)	Raw Reads	Clean Reads	Clean Reads Ratio (%)	Mapped Reads Ratio (%)	Detected Gene Number
C4h-1	150	62058308	61216228	98.64	82.79	12605
C4h-2	150	74317910	73087158	98.34	81.68	12765
C4h-3	150	65623654	64806176	98.75	83.67	12676
D4h-1	150	72355478	71475480	98.78	81.79	12727
D4h-2	150	70127974	69159932	98.62	81.45	12700
D4h-3	150	67394630	66417284	98.55	82.45	12748
L4h-1	150	75659098	74755562	98.81	80.57	12801
L4h-2	150	69452342	68558994	98.71	80.3	12785
L4h-3	150	65264048	64465772	98.78	79.78	12694
C8h-1	150	69453594	68637212	98.82	82.06	12734
C8h-2	150	76623908	75674390	98.76	82.32	12891
C8h-3	150	71397364	70552920	98.82	82.08	12833
D8h-1	150	73407730	72504312	98.77	82.45	12850
D8h-2	150	67833338	67023630	98.81	83.21	12713
D8h-3	150	70832694	69984558	98.8	83.15	12837
L8h-1	150	72237008	71372390	98.8	80.15	12885
L8h-2	150	73850386	72797386	98.57	79.58	12863
L8h-3	150	69607688	68648300	98.62	79.86	12858

**Table 2:** The number of differentially expressed genes (DEGs) for different groups. L4h, live *E. tarda* treatment for 4 h; L8h, live *E. tarda* treatment for 8 h; D4h, dead *E. tarda* treatment for 4 h; D8h, dead *E. tarda* treatment for 8 h; C4h, control group at 4 h of treatment; C8h, control group at 8 h of treatment.

	L4h-vs-C4h	D4h-vs-C4h	L4h-vs-D4h	L8h-vs-C8h	D8h-vs-C8h	L8h-vs-D8h
Upregulated DEGs	81	268	57	121	313	63
Downregulated DEGs	64	119	151	59	175	169
Total DEGs	145	387	208	180	488	232

**Table 3:** Immune-related DEGs in L4h-vs-D4h and L8h-vs-D8h. The “-” symbol before the fold change number indicates downregulation. ns, not significant.

Category and Gene Name	Fold Change	
	L4h-vs-D4h	L8h-vs-D8h
**RIG-I-like receptors**		
Interferon induced with helicase C domain 1 (*IFIH1*)	−2.69	−2.91
DEAD box polypeptide 58 (*DDX58*)	ns	−2.49
DEXH box polypeptide 58 (*DHX58*)	ns	−2.17
**Cytokines**		
Chemokine (C-C motif) ligand 22 (*CCL22*)	−3.89	ns
Chemokine (C-C motif) ligand 3 (*CCL3*)	−2.30	ns
Chemokine (C-C motif) ligand 5 (*CCL5*)	−7.06	−5.08
Chemokine (C-X-C motif) ligand 10 (*CXCL10*)	−3.51	ns
Chemokine (C-C motif) ligand 22 (*CCL2*)	−3.01	–2.34
Chemokine (C-X-C motif) ligand 2 (*CXCL2*)	−2.54	ns
Chemokine (C-C motif) ligand 4 (*CCL4*)	−3.50	ns
Chemokine (C-C motif) ligand 7 (*CCL7*)	−2.91	−2.79
Interleukin 6 (*IL6*)	−11.96	−14.96
Interleukin 27 (*IL27*)	−3.25	−3.25
Interleukin 1 family, member 9 (*IL1F9*)	−2.20	−2.91
Leukemia inhibitory factor (*LIF*)	−4.18	ns
Colony stimulating factor 3 (*CSF3*)	−3.69	−2.59
**Interferon-related genes**		
ISG15 ubiquitin-like modifier (*ISG15*)	−3.81	−4.34
Guanylate binding protein 5 (*GBP5*)	−4.30	−5.75
Guanylate binding protein 2 (*GBP2*)	−4.51	−5.71
Guanylate binding protein 2b (*GBP2b*)	−3.02	−5.63
Guanylate binding protein 7 (*GBP7*)	ns	−3.23
Guanylate binding protein 9 (*GBP9*)	ns	−2.74
Guanylate binding protein 3 (*GBP3*)	ns	−3.75
Interferon-induced protein with tetratricopeptide repeats 3 (*IFIT3*)	−3.06	−4.76
Interferon-induced protein with tetratricopeptide repeats 3b (*IFIT3b*)	−3.30	−4.01
Interferon-induced protein with tetratricopeptide repeats 1 (*IFIT1*)	−4.07	−3.88
Interferon-induced protein with tetratricopeptide repeats 2 (*IFIT2*)	ns	−2.89
Immunity-related GTPase family M member 1 (*IRGM1*)	−2.03	−3.18
Immunity-related GTPase family M member 2 (*IRGM2*)	−2.76	−3.06
Interferon gamma inducible protein 47 (*IFI47*)	ns	−2.92
Interferon regulatory factor 9 (*IRF9*)	−2.13	ns
**Other immune-related genes**		
Myristoylated alanine rich protein kinase C substrate (*MARCKS*)	−6.32	−4.07
Complement component 5a receptor 1 (*C5AR1*)	−2.07	ns
Programmed cell death 1 (*PDCD1*)	−2.01	ns
Heat shock protein 1B (*HSPA1B*)	2.46	2.06
Endothelin 1 (*EDN1*)	−5.53	−3.22
Signal transducer and activator of transcription 2 (*STAT2*)	ns	−2.04
Nitric oxide synthase 2, inducible (*NOS2*)	ns	−2.06

**Table 4:** Summary of top 10 key DEGs based on protein-protein interaction networks.

Gene Name	Description	Number of Protein-Protein Interaction
*CXCL10*	Chemokine (C-X-C motif) ligand 10	29
*IL6*	Interleukin 6	28
*IFIT2*	interferon-induced protein with tetratricopeptide repeats 2	23
*CCL5*	Chemokine (C-C motif) ligand 5	23
*ISG15*	ISG15 ubiquitin-like modifier	21
*IFIH1*	Interferon induced with helicase C domain 1	21
*IFIT1*	Interferon-induced protein with tetratricopeptide repeats 1	21
*CCL2*	Chemokine (C-C motif) ligand 2	19
*DDX58*	DEAD (Asp-Glu-Ala-Asp) box polypeptide 58	19
*IRGM1*	immunity-related GTPase family M member 1	18

**Table 5:** List of primers used for qRT-PCR validation.

Gene Name	Forward Primer (5′-3′)	Reverse Primer (5′-3′)	Amplicon Length (bp)
*CCL2*	TGCTGACCCCAAGAAGGAAT	TGAGGTGGTTGTGGAAAAGGTA	184
*CCL5*	GACACCACTCCCTGCTGCTT	ACACTTGGCGGTTCCTTCG	133
*CXCL10*	CATCCTGCTGGGTCTGAGTG	ACATTCTTTTTCATCGTGGCA	177
*CSF3*	CCAGAGGCGCATGAAGCTAA	GCTCCAGGGACTTAAGCAGG	233
*HSPA1B*	AGAAGGTGCTGGACAAGTGC	AGGCTCCTTTCGGCGG	192
*GBP5*	AGGTCAACGGACCTCGTCTA	CCGGGCCAAGGTTACTACTG	104
*EDN1*	ACCGTATGGACTGGGAGGTT	GGTGAGCGCACTGACATCTA	101
*IFIH1*	CCCAGAAGACAACACAGAATCA	TGGCTCGGGGGATACTCTTT	163
*IFIT1*	AAGGCTGTCCGGTTAAATCC	GAGCTTTGTCTACGCGATGT	190
*ISG15*	GTGCTCCAGGACGGTCTTAC	GACCTCATAGATGTTGCTGTGG	138
*IL27*	CTTCCCAATGTTTCCCTGAC	CGAAGTGTGGTAGCGAGGA	83
*IL6*	GGGAAATCGTGGAAATGAGA	AGGACTCTGGCTTTGTCTTTC	247
*NOS2*	GAGCAACTACTGCTGGTGGT	CGATGTCATGAGCAAAGGCG	178
*GAPDH*	ATTCAACGGCACAGTCAAGG	GATGTTAGTGGGGTCTCGCTC	91

## Supplementary Materials

Supplementary materials can be found at https://www.mdpi.com/1422-0067/20/22/5724/s1.
