# Supplementary material for: A Comparative Analysis of Edwardsiella tarda-Induced Transcriptome Profiles in RAW264.7 Cells Reveals New Insights into the Strategy of Bacterial Immune Evasion
Source: Int J Mol Sci. 2019 Nov 15;20(22):5724. doi: 10.3390/ijms20225724 (PMC6888325; doi:10.3390/ijms20225724)
Supplement: Supplementary file 1 [file ijms-20-05724-s001.pdf]

Supplementary data

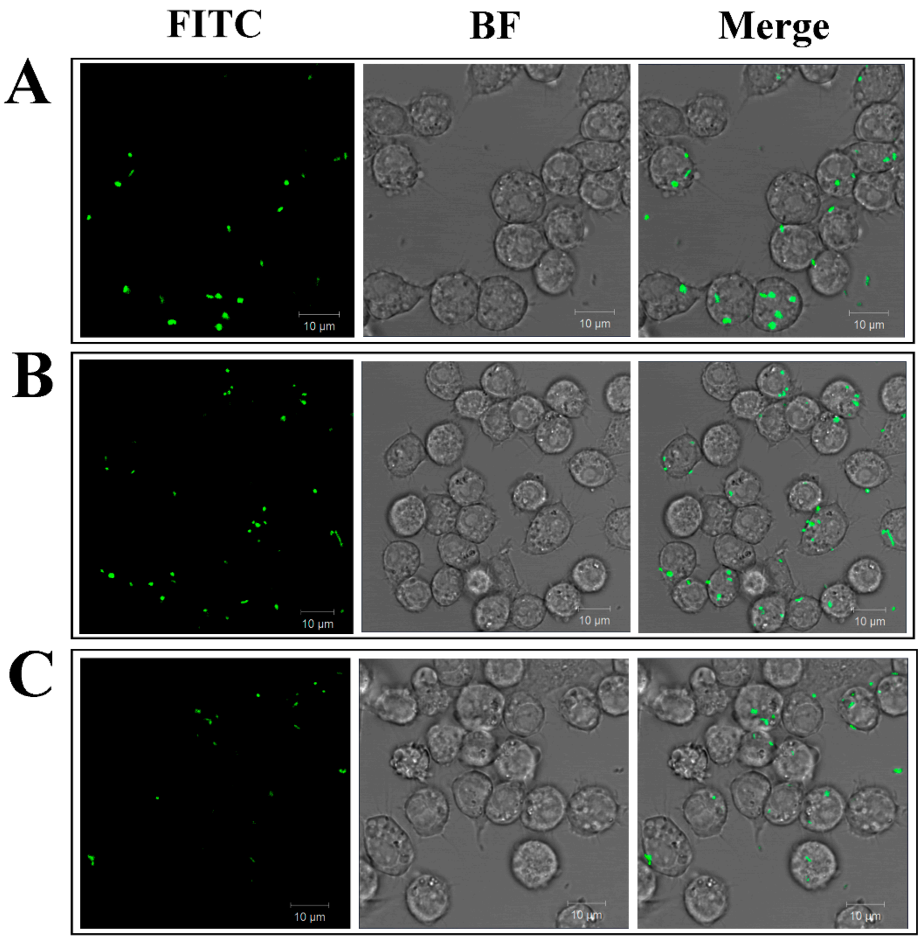

Figure S1. Microscopic observation of RAW264.7 cells treated with inactivated (dead) *Edwardsiella tarda*. RAW264.7 cells were infected with fluorescein isothiocyanate (FITC)-labeled dead *E. tarda* for 2 h and then treated with 100 μg/ml gentamicin for 1 h. The cells were washed and incubated in fresh Opti-MEM containing 20 μg/ml gentamicin for 0 h (A), 4 h (B), and 8 h (C). After incubation at each time point, the cells were observed with a confocal microscope under bright field (BF) and fluorescent light (FITC). The merged image of each panel is shown on the right. Scale bar, 10 μm.

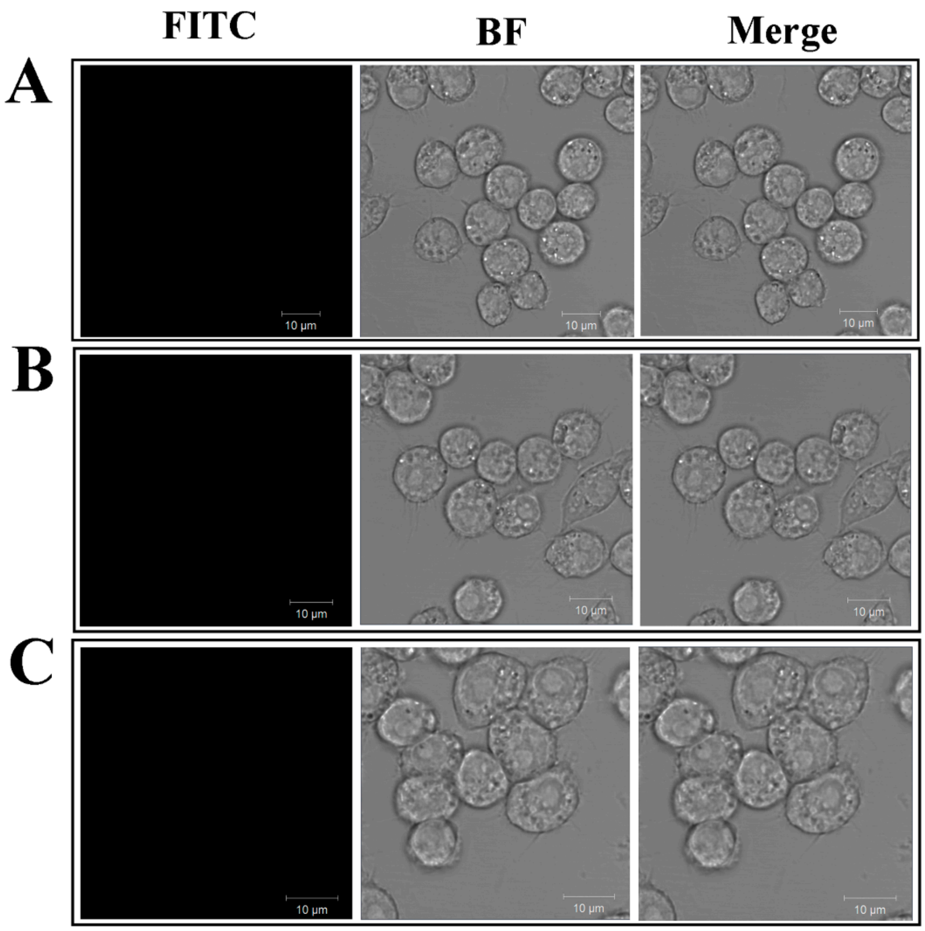

Figure S2. Microscopic observation of untreated RAW264.7 cells. RAW264.7 cells were incubated in Opti-MEM for 2 h and treated with 100 μg/ml gentamicin for 1 h. The cells were washed and incubated in fresh Opti-MEM containing 20 μg/ml gentamicin for 0 h (A), 4 h (B), and 8 h (C). After incubation at each time point, the cells were observed with a confocal microscope under bright field (BF) and fluorescent light (FITC). The merged image of each panel is shown on the right. Scale bar, 10 μm.

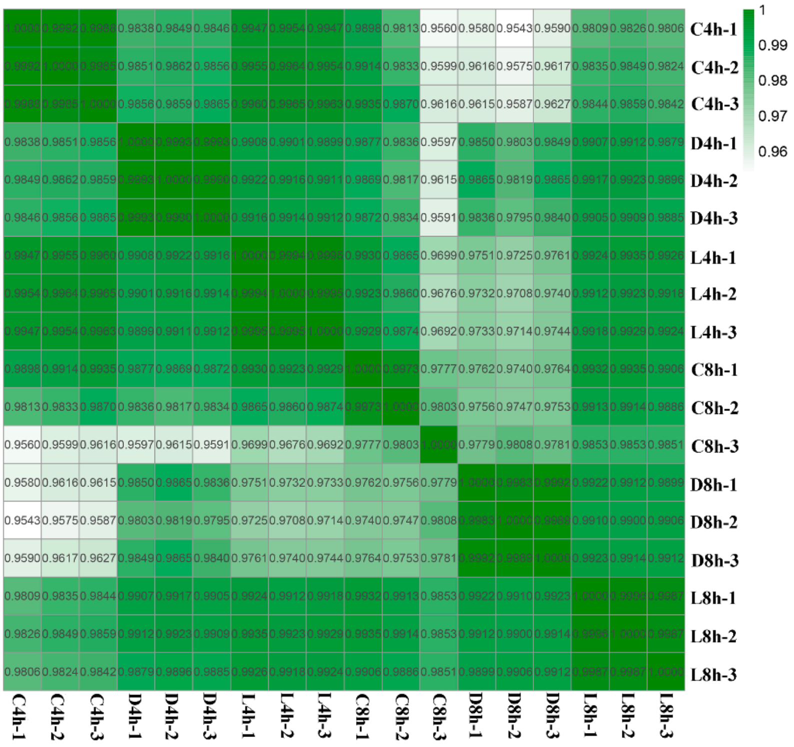

Figure S3. Heat map of correlation between sequencing samples.
